# Vaccination-induced neutralizing antibodies in immunocompetent hosts correlate with protection against rickettsiae

**DOI:** 10.1038/s41541-025-01228-4

**Published:** 2025-08-01

**Authors:** Loka Reddy Velatooru, Jessica Plante, Chen Yi Chu, Garrett Cutchin, Bing Zhu, Nicole Burkhardt, Carsen Roach, Yingzi Cong, Shahid Karim, Yong-Fang Kuo, David H. Walker, Ulrike Munderloh, Rong Fang

**Affiliations:** 1 Department of Pathology, University of Texas Medical Branch, Galveston, TX USA; 2 Department of Microbiology and Immunology, University of Texas Medical Branch, Galveston, TX USA; 3 Department of Entomology, University of Minnesota, St. Paul, MN USA; 4 Division of Gastroenterology and Hepatology, Department of Medicine, Feinberg School of Medicine, Northwestern University, Chicago, IL USA; 5 School of Biological, Environmental, and Earth Sciences, University of Southern Mississippi, Hattiesburg, MS USA; 6 Department of Biostatistics & Data Science, University of Texas Medical Branch, Galveston, TX USA

**Keywords:** Vaccines, Bacteria, Immunology, Vaccines, Live attenuated vaccines

## Abstract

We previously demonstrated that a single-dose immunization with a live-attenuated *Rickettsia parkeri* mutant 3A2 confers complete protection against murine spotted fever rickettsioses. In this study, we investigated whether vaccination-elicited serum antibodies serve as immune correlates of protection against rickettsiae. Immunization of immunocompetent C3H/HeN mice with 3A2 induced a robust and durable level of IgG antibody response, predominantly comprising IgG2a, IgG3, and IgG2b subclasses, and was associated with a significant expansion of CD19^+^ CD45R^+^ IgD^low^ plasma cells. Compared to mock controls, passive transfer of immune sera from 3A2-immunized mice protected C3H-SCID mice from *R. parkeri* challenge via multiple inoculation routes, including i.v., i.d. and i.d. plus tick saliva. Strikingly, serum antibodies of 3A2-immunized mice significantly reduced the number of *R. parkeri* plaques in vitro, indicating direct neutralizing activity. Collectively, our findings demonstrate that vaccination-induced serum IgG antibodies correlate with protection against rickettsial infection and possess direct neutralizing effects on rickettsiae.

## Introduction

*Rickettsia* is a genus of Gram-negative, obligately intracellular α-proteobacteria. Based on their distinctive antigenic profiles, particularly serological features^1^, rickettsiae are traditionally divided into two groups: the spotted fever group (SFG) and the typhus group (TG). Most SFG rickettsiae pathogenic to humans are transmitted by ticks, which are responsible for transmitting almost 95% of vector-borne pathogens reported in the United States (US)^2^. Since 2004, reported cases of tick-borne diseases (TBDs) in the US have nearly tripled, highlighting including tick-borne rickettsioses (TBRs), as a growing threat to public health^3^. *Rickettsia parkeri* is a species in the SFG that causes a mild infection known as American boutonneuse fever or *Rickettsia parkeri* rickettsiosis, characterized by the formation of an eschar at the tick bite site^4^. Given the relatively low virulence and pathogenicity, *R. parkeri* has garnered interest as, at least part of, a live-attenuated candidate vaccine for TBRs. *R. rickettsii* is the etiological organism of Rocky Mountain spotted fever (RMSF), which is the world’s deadliest tick-borne disease^3^. Typically, RMSF initiates with a sudden onset of fever, headache, and rash after exposure to infected ticks or dogs that carry infected ticks. The illness can progressively worsen in 7–10 days into respiratory failure and seizures or even death in untreated or inappropriately treated patients^5^. Case fatality rates of RMSF can range from 5 to 20% within the United States and up to 50% in Brazil and Mexico^6–8^. Due to their potentially severe infection outcomes and possible transmission by aerosolized particles^9^, there is also a concern about the use of rickettsiae as a bioweapon^10^. Vaccines are the most effective means to prevent infectious diseases. However, for most of the TBDs, including SFRs, no FDA-licensed vaccine is available. Thus, developing a vaccine against TBRs is a key strategy for protecting high-risk populations and a high priority to address the threat to public health posed by these diseases.

Historically, several vaccines against rickettsiae have been attempted, including whole killed bacteria, subunit vaccines and live-attenuated bacteria, most of which were developed through empirical research without a thorough understanding of vaccine-induced immunity^11–13^. Whole killed *Rickettsia* has not been widely used because it only provides partial protection against RMSF^14,15^. Most of the studies on subunit vaccines against rickettsioses have focused on the outer membrane protein A (OmpA), or surface cell antigen 0 (Sca0) or OmpB (Sca5), fragments of which provide relatively promising protection against rickettsial infection in animals^16–20^. We have recently reported that a single-dose immunization with a *R. parkeri* mutant 3A2, which expresses a defective phage integrase family protein, stimulates protective immunity against multiple murine rickettsioses^12^. Another mutant of *R. parkeri*, with a defective OmpB or Sca5, protects mice from the establishment of eschar, a major pathological lesion of *R. parkeri* rickettsiosis^21^.

Successful development of a licensed vaccine against rickettsiae is hindered by the gaps in our understanding of the essential elements of host immune responses that account for vaccine-induced protection, as well as insufficiently explored methodologies for assessing diverse vaccine candidates. Although CD8^+^ T cells are considered to play a critical role in host protection against rickettsiae^22^, most studies on vaccine-induced protection against SFR have primarily focused on antibody responses, particularly those recognizing recombinant OmpA or OmpB. *Rickettsia*-specific polyclonal and monoclonal antibodies provide protection involving mechanisms of Fc-dependent phagolysosome and complement-mediated killing of rickettsiae in phagocytes and blood, respectively^17,20–25^. The most abundant isotype in healthy human serum, immunoglobulin G (IgG), consists of four subclasses. Although IgG is the primary antibody isotype circulating within individuals who have been exposed to or infected with rickettsiae, only a few subclasses of IgG antibodies to SFR have been reported previously^12,26^. A profile of IgG subclasses induced by the candidate vaccine against rickettsiae is currently lacking but clearly needed. Additionally, it remains elusive whether serum antibodies elicited by the vaccine candidates effectively protect hosts, particularly those challenged by routes other than intravenous inoculation, the experimental model upon which most fatal murine rickettsioses are established^27^. Neutralizing activity mediated by serum antibodies has been utilized as a standard to predict and evaluate the protective efficacy of candidate vaccines against various intracellular pathogens such as viruses and *Chlamydia*^28,29^. An in vitro method as a benchmark for evaluating the vaccine candidates against rickettsiae has not yet been clearly described. Thus, we investigated whether rickettsiae-specific IgG antibodies in vaccine-induced immune serum are capable of directly inhibiting the infectivity of rickettsiae to mammalian host cells.

To this end, we hypothesize that antibodies in sera, induced by a live-attenuated vaccine candidate, are crucial for conferring host protection against rickettsioses, at least through direct inhibition of the infectivity of rickettsiae. We demonstrated that passive transfer of *R. parkeri* mutant 3A2 (*R. parkeri* 3A2 or 3A2) immune sera provided protection against lethal infection of *R. parkeri* in mice challenged via various inoculation routes, including intravenous, intradermal and intradermal plus tick saliva. Strikingly, we revealed that the serum antibodies induced by vaccination with *R. parkeri* 3A2 directly blocked the interactions of rickettsiae with host cells, namely neutralization. Therefore, our present studies, for the first time, demonstrated the in vitro neutralization of rickettsiae by vaccine-induced serum antibodies, highlighting the critical role of antibodies in mediating vaccine-conferred protection against rickettsiae.

## Results

### A single-dose of *R. parkeri* 3A2 elicited a durable IgG antibody response specific against *R. parkeri*

**Fig. 1:**
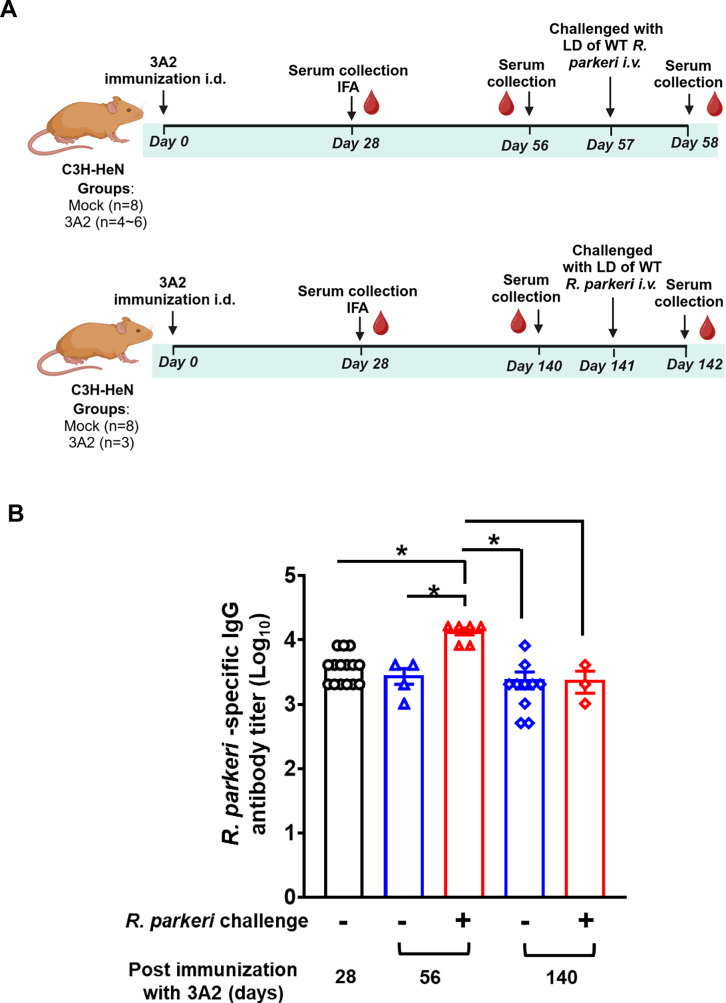
*R. parkeri* 3A2 elicited a durable and saturated level of IgG antibodies against *R. parkeri.* **A** Schematic illustration of mouse immunization, rickettsial challenge and serum collection. C3H/HeN mice were i.d. immunized with live-attenuated *R. parkeri* 3A2 at a single-dose of 10^3 copies of CS gene per mouse. On days 28, 56, and 140 post immunization, serum was collected. On days 57 and 141 post immunization, respectively, mice were challenged with a lethal dose (LD) of *R. parkeri* and euthanized on day 1 post challenge. **B** Serum was collected at the indicated time points. The titers of IgG antibodies specific against *R. parkeri* in sera were determined by indirect immunofluorescence assay (IFA). Mock-immunized mice served as a negative control. No significant antibody titer was detected in the control group. Each group included 3~10 mice. ns, not statistically significant. *p < 0.05. Results represent two independent experiments with consistent results.

A durable antibody response is crucial for the evaluation of an effective vaccine and vaccine-conferred protection. To this end, we intradermally (i.d.) immunized C3H/HeN mice with a single-dose of *R. parkeri* 3A2 and monitored the levels of specific IgG antibody response for up to 140 days by indirect immunofluorescence assay (IFA). In line with our previously published studies, *R. parkeri* 3A2 immunization mounted a significantly elevated IgG antibody response compared to the mock-immunized group as early as on day 28 post-immunization (Fig. 1A, B). The titer of IgG antibodies against wild type (WT) *R. parkeri* (or *R. parkeri*) did not show a significant reduction in sera of *R. parkeri* 3A2-immunized mice collected on days 56 and 140 post immunization compared to those on day 28 post immunization (Fig. 1A, B), highlighting a durable antibody response.

On day 56 post immunization, lethal challenge significantly increased the titer of IgG antibody compared to the pre-challenge, suggesting that the IgG antibody response is not completely saturated. Interestingly, on day 141 post immunization, upon lethal challenge, the titers of IgG antibody did not significantly alter compared to those before challenge (Fig. 1), suggesting that the IgG antibody response is saturated at this time. Therefore, a single-dose immunization with *R. parkeri* induced a durable and saturated IgG antibody response.

### The isotype profile of protective antibodies induced by a single-dose immunization with *R. parkeri* 3A2

**Fig. 2:**
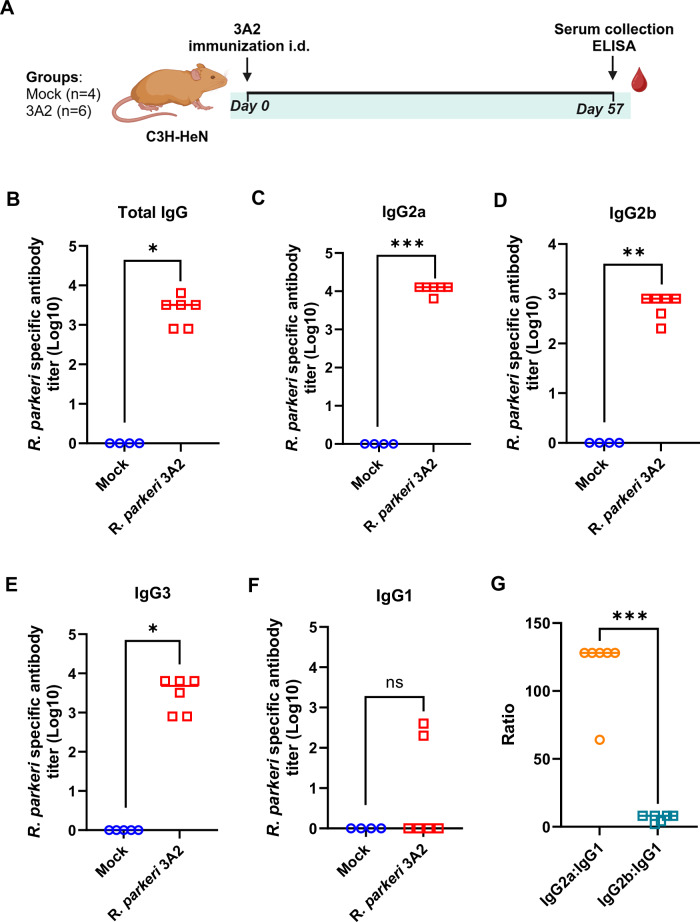
Subclass profile of IgG antibodies specific against *R. parkeri* in sera of *R. parkeri* 3A2-immunized mice. **A** Schematic diagram of vaccination of mice followed by collection of serum specimens. C3H/HeN mice were immunized with a single-dose of *R. parkeri* 3A2 i.d. Mice immunized the PBS served as mock controls. Sera were collected on day 57 post immunization. The titers of *R. parkeri*-specific total IgG (**B**), IgG2a (**C**), IgG2b (**D**), IgG3 (**E**), and IgG1 (**F**) antibodies in immune sera were determined by ELISA as described in the corresponding section of Methods. **G** The ratios of titers of IgG2a:IgG1 and IgG2b:IgG1 were calculated. (Each group includes 4~6 mice. *p < 0.05, **p < 0.01, ***p < 0.001 (compared with the mock group). ns, not statistically significant.

To further study the antibodies induced by vaccination with *R. parkeri* 3A2, the subclasses of serum antibodies specific against *R. parkeri*, including IgG1, IgG2a, IgG2b, IgG3, and IgA, were characterized. C3H/HeN mice were immunized intradermally with either *R. parkeri* 3A2 or PBS (Fig. 2A). Serum samples collected on day 57 post immunization were analyzed for *R. parkeri*-specific IgA and IgG subtypes using ELISA. Mock-immunized mice did not produce any detectable IgG antibodies reactive against *R. parkeri* (Fig. 2B). Mice immunized with *R. parkeri* 3A2 displayed a significant isotype switch in their IgG responses, with production of IgG2a (Fig. 2C), IgG2b (Fig. 2D), and IgG3 (Fig. 2E) subclasses. Interestingly, no significant IgG1 and IgA antibody responses were detected (Fig. 2F and supplementary Fig. 1). Furthermore, the ratio of IgG2a/IgG1 was significantly greater than that of IgG2b/IgG1 (Fig. 2G). Notably, *R. parkeri* 3A2 immunization elicited a prominent IgG2a and IgG3 response compared to mock-immunized mice (Fig. 2), which is characteristic of a Th1-driven immune response.

### A single-dose immunization with *R. parkeri* 3A2 triggered a significant expansion of antibody-producing plasma cells

**Fig. 3:**
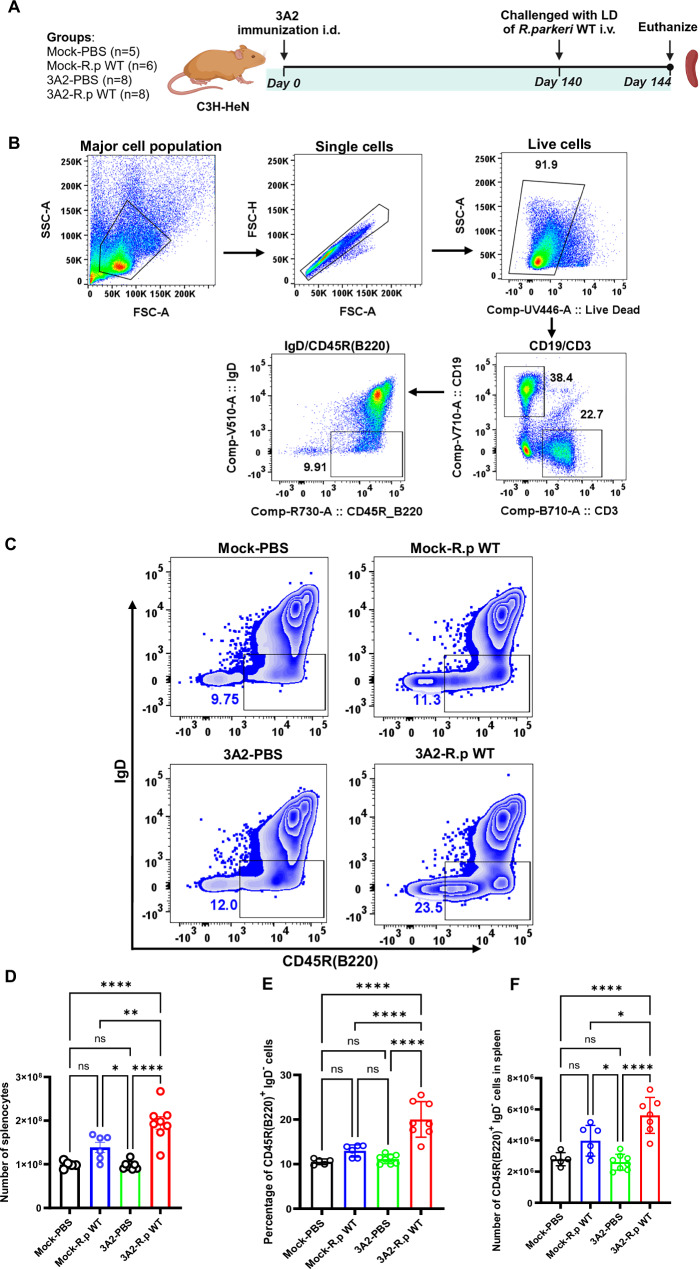
Immunization with *R. parkeri* 3A2 elicited a significantly expanded plasma cell population in spleen. **A** Schematic illustration of experimental design and timeline. C3H/HeN mice were immunized with *R. parkeri* 3A2 at a single-dose of 10^3 copies of CS gene per mouse i.d. After 140 days of immunization, mice were challenged with a lethal dose of *R. parkeri* i.v. PBS immunized or challenged mice served as negative controls. On day 4 post challenge, mice were humanely euthanized, and spleens were collected. **B** Representative dot plots of the gating strategies on the live CD19^+^ CD45R/B220^+^ B cells. **C** FACS plots displaying the percentage of CD19^+^ CD45R/B220^+^ IgD^low^ class switching B cells. The frequencies of splenocytes **D**, percentages and numbers of plasma cells (**E** and **F**) in spleen were determined by flow cytometric analysis. *p < 0.05, **p < 0.01, ****p < 0.0001. ns, not statistically significant. Results represent two independent experiments with consistent results.

To investigate the B-cell immune response associated with the production of IgG antibodies, we analyzed class-switching B cells in the spleens of *R. parkeri* 3A2-immunized mice by flow cytometric analysis in response to intravenous inoculation with *R. parkeri* (Fig. 3A). A single-dose immunization with 3A2 did not significantly change the frequency of splenocytes compared to the controls (Fig. 3B–D). Upon i.v. lethal challenge with *R. parkeri*, 3A2 immunized mice showed a significantly greater number of splenocytes compared to mock-immunized mice (Fig. 3B–D). Splenic antibody-producing plasma cells or class-switching B cells were stained as CD19^+^ CD45R/B220^+^ IgD^low^ (Fig. 3B, C). Interestingly, compared to control mouse groups, mice immunized with 3A2 then challenged with a lethal dose of *R. parkeri* showed significantly expanded percentage and number of CD19^+^CD45R/B220^+^ and IgD^low^ antibody-secreting plasma cells (Fig. 3E, F).

### *R. parkeri* 3A2 was safe in immunocompetent hosts but demonstrated adverse outcomes in immunocompromised hosts

**Fig. 4:**
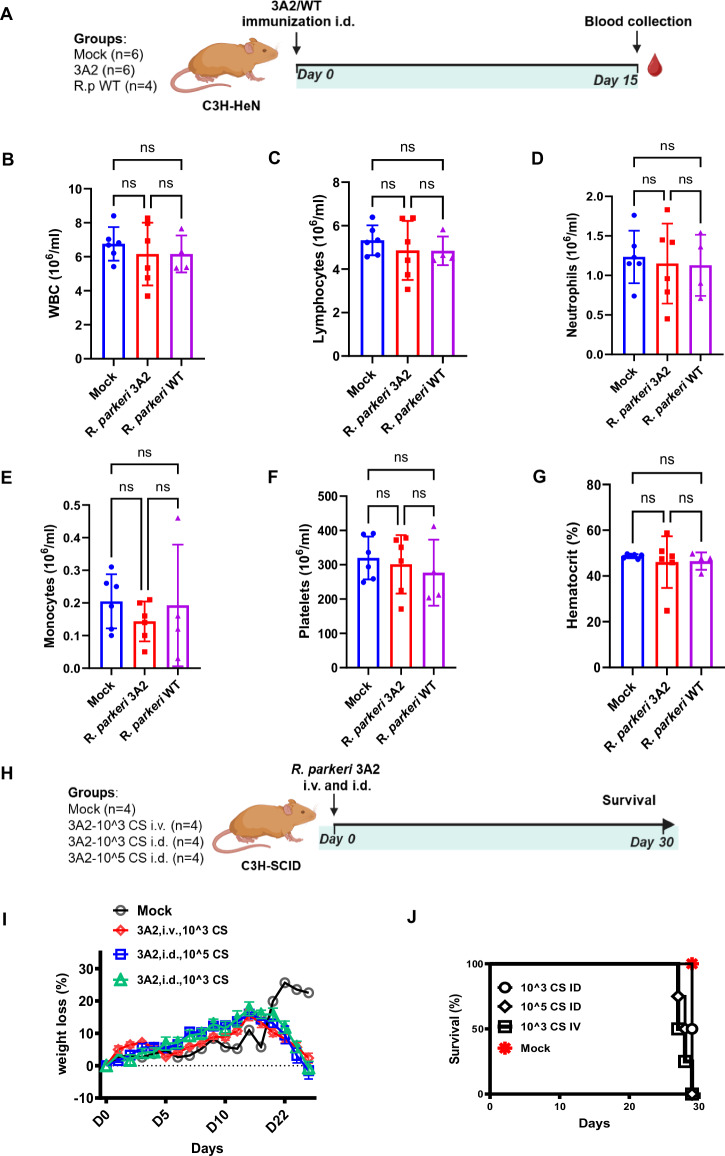
Comprehensive evaluation of the safety of *R. parkeri* 3A2 as a vaccine candidate. **A** Schematic illustration of experimental design of immunocompetent hosts. C3H/HeN mice were immunized with *R. parkeri* 3A2 i.d. On day 15 post immunization, blood was collected for hematological analysis to evaluate the frequency of leukocytes (**B**), lymphocytes (**C**), neutrophils (**D**), monocytes (**E**), platelets (**F**) and percentage of hematocrit (**G**). **H** Schematic illustration of experimental design of immunocompromised hosts. C3H-SCID mice were inoculated with *R. parkeri* 3A2 using indicated doses and routes i.d., intradermally; i.v., intravenously. Mock-immunized mice served as controls. Mice were monitored for weight loss (**I**) and survival (**J**). Each group included 4 mice. ns, not statistically significant.

To further evaluate the safety profile of *R. parkeri* 3A2 as a live-attenuated vaccine candidate, we analyzed different components in blood of immunized C3H/HeN mice during the early phase of immunization (Fig. 4A). We did not find any significant expansion of inflammatory leukocytes (Fig. 4B), lymphocytes (Fig. 4C), neutrophils (Fig. 4D), monocytes (Fig. 4E), platelets (Fig. 4F) and red blood cells (Fig. 4G), suggesting that immunization with live-attenuated 3A2 did not induce any detectable levels of pro-inflammatory response.

Individuals with impaired immunity also need preventive approaches against rickettsial diseases. To determine whether the live-attenuated vaccine candidate against rickettsiae is appropriate for immunocompromised hosts, we inoculated C3H-SCID (or SCID) mice with a single-dose of *R. parkeri* 3A2 as indicated (Fig. 4H). Upon intradermal inoculation with *R. parkeri* 3A2 at a dose previously shown to confer protection against two fatal rickettsioses^12^, C3H-SCID mice exhibited no signs of illness or significant weight loss until day 20 post infection (p.i.) (Fig. 4I). After day 20 of infection, C3H-SCID mice progressively showed illness and weight loss and succumbed to the infection on day 28–30 p.i. (Fig. 4I, J). To further investigate the safety profile of *R. parkeri* 3A2, we inoculated C3H-SCID mice with a high dose i.d. and intravenously (i.v.) As shown in Fig. 4I, J, regardless of the dose or inoculation route, C3H-SCID mice showed a very similar illness pattern and weight loss and all succumbed to the infection on days 28 ~ 30 p.i. These results clearly indicate that despite its robust immunogenicity and protective efficacy in immunocompetent hosts, the live-attenuated vaccine candidate against rickettsiae may not be suitable for severely immunocompromised hosts.

### Passive transfer of *R. parkeri* 3A2-immune sera conferred protection against i.v. challenge in mice

**Fig. 5:**
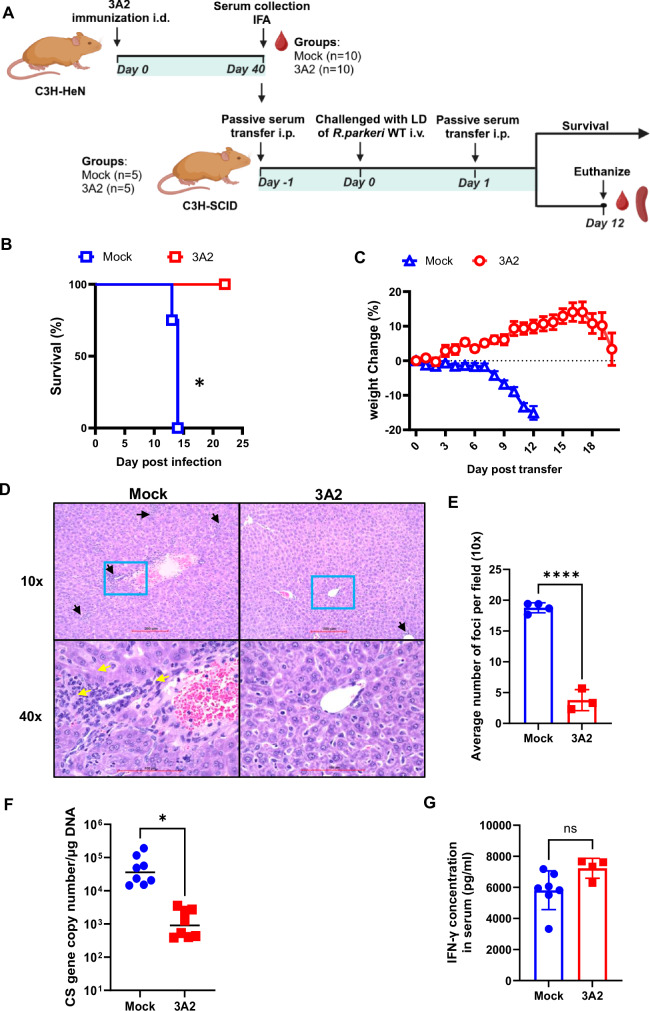
Passive transfer of *R. parkeri* 3A2-immune sera protected severely immunocompromised C3H-SCID mice from i.v. lethal challenge with *R. parkeri.* **A** Schematic illustration of experimental design and timeline. C3H/HeN mice were immunized with *R. parkeri* 3A2 as described in Methods section. Sera were collected after 40 days of immunization. C3H-SCID mice were intraperitoneally (i.p.) passively transferred with immune or control mouse sera one day prior to and after challenge with a lethal dose of *R. parkeri* i.v. Mice were monitored for survival (**B**) and weight loss (**C**). In parallel, a group of C3H-SCID mice were humanely euthanized on days 12 p.c. and livers were collected for histopathological analysis (**D**). The black arrows show inflammatory foci, and yellow arrows point to the infiltrated inflammatory cells and perivascular inflammation. Area under blue square imaged at 40x magnification (**D**). The average number of inflammatory foci obtained from ten fields for each H&E-stained liver sample (10x) (**E**). Bacterial burden in tissues by quantitative real-time PCR amplifying numbers of CS genes (**F**). **G** After mice were euthanized, sera were collected for assessing the concentration of IFN-γ by ELISA. Each group included 4 ~ 8 mice. *p < 0.05, ****p < 0.0001. ns, not statistically significant.

To determine the contribution of antibodies to vaccine-induced host protection against rickettsiosis, we collected and passively transferred the pooled sera of *R. parkeri* 3A2-immunized C3H/HeN mice to B- and T- cell deficient C3H-SCID mice one day before and one day after lethal challenge with *R. parkeri* i.v. (Fig. 5A). Following the passive transfer of serum, none of the recipient mice showed any signs of illness during the first three days of infection. Infected C3H-SCID mice that received mock-immunized sera began to show weight loss on day 8 post challenge (p.c.) and succumbed on days 13 and 14 p.c. (Fig. 5B, C). In contrast, all C3H-SCID mice passively transferred with *R. parkeri* 3A2 immune sera showed progressively increased body weight and significantly prolonged survival after a lethal challenge with *R. parkeri* compared to the animals that received mock-immune serum (Fig. 5B, C).

The *R. parkeri* 3A2-immune sera protected C3H-SCID mice from developing pathological lesions in the livers following a lethal challenge with *R. parkeri*. Histopathological analysis of the livers of C3H-SCID mice that received mock-immune sera revealed marked pathological damage, characterized by numerous inflammatory foci and inflammatory cell infiltrations surrounding the vessels, comprising both lymphocytes, possibly NK cells, and other mononuclear cells (Fig. 5D). In contrast, mice passively transferred 3A2 immune sera had few pathological changes, with only a minimal number of infiltrated cells. The average number of foci per field demonstrated a significant decrease in inflammatory cell infiltration in mice that received 3A2 immune sera compared to controls (Fig. 5E). Furthermore, the concentrations of rickettsiae in livers of C3H-SCID mice that received mock-immunized sera were significantly greater compared to those that received *R. parkeri* 3A2 immune sera (Fig. 5F). Interestingly, no significant difference was found in concentrations of serum IFN-γ between C3H-SCID mice that received 3A2 immune sera and those that received mock-immune sera (Fig. 5G), highlighting the critical role of serum antibodies in protecting host against lethal challenge with *R. parkeri*. These results indicate that serum antibodies are an immune correlate of vaccine-conferred protection against rickettsiae.

### Passive transfer of *R. parkeri* 3A2-immune sera provided significant protection against i.d. challenge with tick saliva

**Fig. 6:**
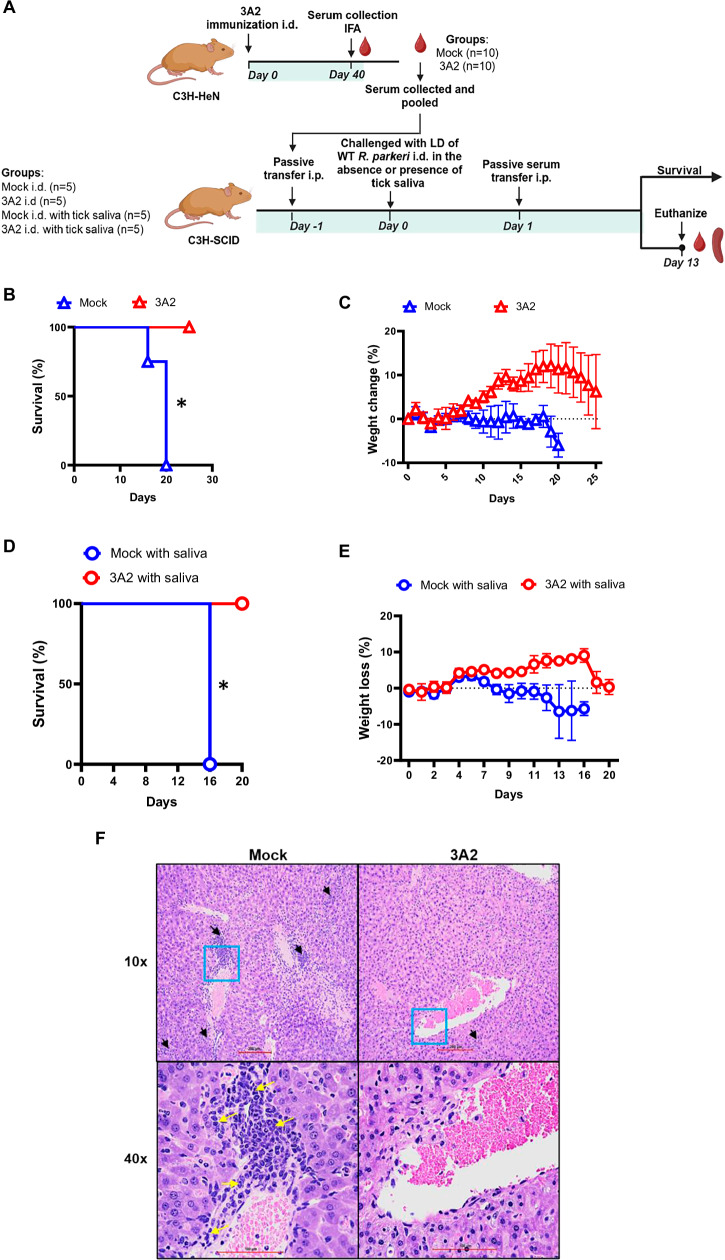
Passive transfer of sera of 3A2-immunized mice provided protection to severely immunocompromised SCID mice against i.d. lethal challenge of rickettsiae with or without tick saliva. **A** Schematic illustration of experimental design and timeline. C3H/HeN mice were immunized with *R. parkeri* 3A2 and sera were collected as described in the Methods. Sera were collected after 40 days of immunization with *R. parkeri* 3A2 or PBS. C3H-SCID mice were passively transferred i.p. with either pooled immune or control sera followed by inoculation with a lethal dose of *R. parkeri* via i.d. or i.d. plus tick saliva. Mice were monitored daily for survival and weight loss (**B**, **C**, **D**, **E**). In parallel, a group of i.d. challenged C3H-SCID mice were humanely euthanized on days 13 p.c., and livers were collected for histopathological analysis. The black arrows show inflammatory foci, and yellow arrows point to the inflammatory cells infiltrated in the foci of perivascular lesion. Area with the blue square imaged at 40x magnification (**F**). Each group included 4 mice. *, *p* < 0.05.

*R. parkeri* is transmitted to humans by the Gulf Coast tick, *Amblyomma maculatum*. To investigate whether vaccination-induced serum antibodies are potentially protective against natural infection with rickettsiae, we challenged naive C3H-SCID mice that received 3A2 immune sera with *R. parkeri* i.d., with or without the saliva of *Amblyomma maculatum* (Fig. 6A). Sera from mock-immunized C3H/HeN mice served as controls. Upon intradermal challenge, all C3H-SCID mice that received mock-immune sera showed weight loss and succumbed to infection on days 16 ~ 20 p.c. (Fig. 6B, C). In contrast, passive transfer of *R. parkeri* 3A2 immune sera provided 100% protection for almost 4 weeks to C3H-SCID mice i.d. lethally challenged with *R. parkeri* (Fig. 6B, C).

When C3H-SCID mice were challenged with a lethal dose of *R. parkeri* containing tick saliva, all animals transferred with mock-immune sera started to lose weight on day 11 p.c. and succumbed to infection on day 16 p.c. (Fig. 6D, E). Passive transfer of *R. parkeri* 3A2 immune sera conferred protection against i.d. lethal challenge, which was associated with ameliorated pathological changes in the liver (Fig. 6F), similar to observations in animals challenged with *R. parkeri* i.v.. All C3H-SCID mice passively transferred with *R. parkeri* 3A2 immune sera were protected compared to mice that received mock-immune sera, strongly indicating that 3A2-induced serum antibodies are a mechanism of protection against *R. parkeri* rickettsiosis in mice across different inoculation routes.

### *R. parkeri* 3A2-induced antibodies possessed neutralizing activity against *Rickettsia*

**Fig. 7:**
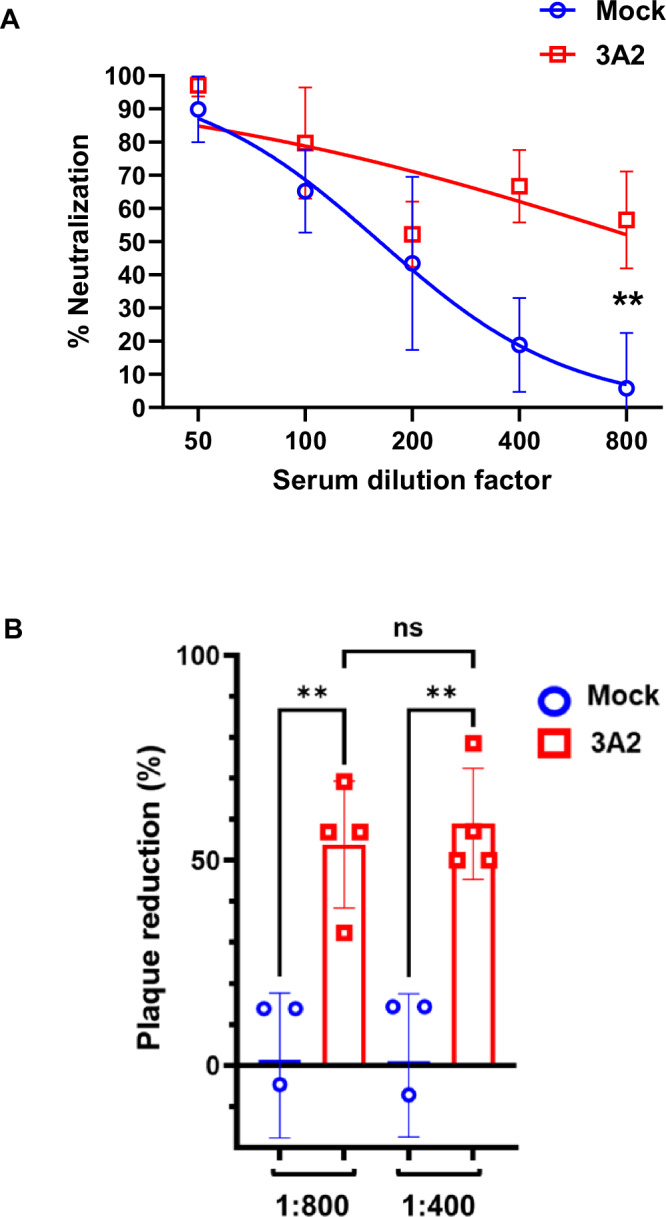
Immunization with *R. parkeri* 3A2 elicited serum antibodies with neutralizing activity against *R. parkeri.* C3H/HeN mice were immunized with a single-dose of *R. parkeri* 3A2 i.d. Sera were collected after 8 weeks of immunization. Pooled sera were heat-inactivated and diluted as indicated. **A** Plaque inhibition rate of *R. parkeri* by neutralizing antibodies in serum as measured by plaque reduction neutralization assay. Symbols represent the mean of four replicates while error bars represent the standard deviation. Smoothed lines represent the fitted curve. Results shown are representative of three independent experiments. **B** The percentage of plaques inhibited by incubation with *R. parkeri* 3A2 immune sera calculated in comparison to mock-sera at two dilutions as indicated. **p < 0.05. ns, not statistically significant. Results represent three independent experiments with consistent results.

To investigate how *R. parkeri* 3A2 immune sera protected immunocompromised hosts against lethal infection, we compared the rickettsial neutralization mediated by immune sera to that of mock serum. As shown in Fig. 7A, *R. parkeri* 3A2 vaccination induced significantly higher levels of neutralizing antibody than mock vaccination, as evidenced by the higher serum dilution required to achieve 50% neutralization (547.3 vs 235.2, *p* = 0.0153) and a comparison of the neutralization curves (*p* = 0.0497). Furthermore, at a dilution of 1:800, sera of *R. parkeri* 3A2-immunized mice reduced the infectivity of *R. parkeri* in Vero cells by 53.85% ± 0.154 whereas sera from the mock-immunized group only reduced infectivity by 0% ± 0.176 (Fig. 7B). The potency to neutralize *R. parkeri* in vitro also differed significantly between *R. parkeri* 3A2- and mock-immune sera at a dilution of 1:400 (58.93% ± 0.135 vs 0% ± 0.175). These results indicated that IgG antibodies in sera from *R. parkeri* 3A2-immunized mice significantly reduced the quantity of infectious *R. parkeri* entering Vero cells, suggesting antibody-mediated neutralization^30–33^.

## Discussion

In this study, we provided evidence that vaccine-induced protection against *R. parkeri* infection correlated with immunization-elicited neutralizing antibodies. Vaccination-induced antibodies not only reduced the rickettsial load in tissues but also ameliorated pathological lesions without altering circulating type I effector cytokines, highlighting the importance of serum antibodies in protecting the mammalian hosts from rickettsial infection. Interestingly, antibodies in immune sera protected animals challenged with a lethal dose of rickettsiae via the routes closely mimicking the natural transmission, offering insights into promising strategies for vaccine development. Importantly, to our knowledge, this is the first report to reveal that vaccination-induced serum antibody mediates neutralization of rickettsiae, which may serve as a rigorous predictive marker of vaccine-elicited protective immunity in vitro.

A comprehensive understanding of the antibody profile and the mechanisms through which antibodies mediate vaccine-induced protection is critical for the optimal design of a successful vaccine^30^. Vaccines against intracellular bacterial pathogens elicit antibodies that primarily confer protection by neutralizing a pathogen or toxin, activating the complement system to lyse bacterial cells, and enhancing phagocytosis of organisms by phagocytic cells (opsonization)^31,34^.

Previously, the potential molecular basis of antibody-mediated rickettsial killing has been evaluated in various types of mammalian host cells. Polyclonal and monoclonal antibodies to *R. conorii* or rickettsial outer membrane proteins enhance the adherence and phagocytosis of *R. conorii* by endothelial cells and macrophages, likely through the binding of the antibodies’ Fc fragments to Fc receptors expressed on these cells^23^. Indeed, the enhanced phagocytosis of rickettsiae was inhibited by normal serum containing Fc fragments of antibodies that are not specifically reactive against rickettsiae. Furthermore, *R. conorii*-specific antibodies and an anti-OmpB monoclonal antibody inhibit the escape of rickettsiae from the phagosomes and promote phagolysosomal killing of rickettsiae, with the involvement of nitric oxide, reactive oxygen intermediates, and l-tryptophan starvation^23^. In addition, the F(ab′)2 fragments of a protective monoclonal antibody reacting with the passenger domain of OmpB fail to prevent the invasion of rickettsiae into Vero cells but reduce the survival of rickettsiae in the blood, through complement-mediated killing^20^.

Neutralizing IgG antibodies represent a key effector component of the humoral immune response elicited by effective vaccines against invading pathogens^31,32^. Antibody neutralization is typically assessed via an in vitro assay in which neutralizing antibodies block pathogen entry into target cells^33^. In our study, we evaluated the neutralizing activity of serum antibodies induced by *R. parkeri* 3A2 vaccination using heat-inactivated serum, thereby excluding complement-mediated effects. Thus, the observed significant reduction in the quantity of *R. parkeri* by incubation with *R. parkeri* 3A2 immune sera indicates a complement-independent mechanism. Furthermore, we quantified infectious rickettsial particles entering Vero cells using a plaque assay. Notably, Vero cells lack Fc receptors^35^, indicating that the reduced number of infectious rickettsiae is due to direct blockade of *R. parkeri* entry by antibodies, most likely mediated via the antigen-binding (Fab) region^31^. These findings provide the first evidence of neutralizing antibody activity in serum induced by vaccination against *R. parkeri*. Nonetheless, the precise mechanisms by which vaccination-elicited antibodies neutralize rickettsiae remain to be elucidated, particularly concerning direct evidence of their capacity to inhibit or block rickettsial binding to host cells. Further in vitro and in vivo studies are warranted to elucidate these mechanisms and to characterize the additional effector functions of antibodies induced by vaccine against rickettsiae. Such insights will accelerate the identification of candidate antigens for subunit vaccine development against rickettsioses.

To study the protective efficacy of vaccination-induced antibodies against the natural transmission of tick-borne rickettsioses, we employed a passive transfer approach in severely immunocompromised C3H-SCID mice, thereby avoiding the effects from the endogenous elements of adaptive immune response in immunocompetent hosts^36–39^. Previously, passively administered polyclonal antibodies to OmpA or OmpB protect guinea pigs against *R. rickettsii* and confer protection in C3H-SCID mice against a lethal infection with a closely related organism^16–20^. In our studies, C3H-SCID mice receiving mock immune sera succumbed to a lethal dose of *R. parkeri* administered i.v., i.d. or i.d. with tick saliva. In contrast, passive transfer of serum from mice immunized with *R. parkeri* 3A2 conferred complete protection against *R. parkeri* irrespective of infectious routes. Although the survival of animals with i.d. challenge differed slightly from those with i.d. challenge plus tick saliva, differences were not statistically significant. Nevertheless, these findings highlight the protective potential of vaccine-induced antibodies in combating natural *R*. *parkeri* infections. Notably, as serum was passively transferred both prior to and following the *R. parkeri* challenge, investigations of the therapeutic potential of *R. parkeri* 3A2-induced antibodies are needed. Moreover, the transferred sera may contribute to protective immunity against rickettsiae through mechanisms beyond neutralizing rickettsiae *in* *vivo*, potentially involving complement activation, Fc-receptor-mediated opsonization and antibody-dependent cellular cytotoxicity^40^. Our previous publication demonstrated that *R. parkeri* 3A2 confers protection in *R. conorii*-infected C3H/HeN mice^12^, a validated experimental model of RMSF^27^. Thus, we propose that *R. parkeri* 3A2-induced antibodies provide protection against *R*. *rickettsii* infection. Given that *R. parkeri* typically causes mild disease in humans^4^, further investigation is warranted to evaluate the protective efficacy of *R. parkeri* 3A2-induced serum antibodies against rickettsial species heterologous to *R*. *par*k*eri*, such as *R. rickettsii* and *R. conorii*, that cause potentially life-threatening diseases in humans.

Different isotypes and subclasses of immunoglobulin may have varying effector functions and translational potentials^41,42^. Although pre-existing IgM in conjunction with the complement system contributes to innate immune responses against invading rickettsial pathogens^43^, rickettsial infection-induced IgM antibodies are known to exhibit low specificity^44^. Thus, we focused on analyzing the subclasses of IgG. To our knowledge, this is one of the first reports that vaccination induced serum *Rickettsia*-specific IgG3 antibodies. IgG1 and IgG3 are the most efficient activators of the classical complement pathway^45^ and the involvement of the complement system in the immune response against rickettsiae has been clearly demonstrated^43^. In line with these findings, we found that IgG3 was among the most abundant subclasses of IgG antibodies specific against *R. parkeri* in vaccinated animals, highlighting the potential importance of IgG3 antibodies and possibly the antibody-dependent complement system in driving host protective immunity against rickettsiosis. High levels of circulating IgG2a and IgG2b, coupled with undetectable levels of IgA and IgG1 antibodies, suggest that a dominant Th1-immune response plays an essential role in providing protection against *R. parkeri* infection, highlighting its potential in vaccine-induced immunity.

Although a live-attenuated vaccine is highly efficient and induces long-lasting and broad immune responses, the safety is always a significant concern. We tracked IgG titers for more than five months without observing a significant decline in the humoral responses, suggesting that our vaccine candidate induces a robust and durable IgG antibody response specific against rickettsiae. Although our work focused on serum antibodies, we propose that a vaccine-induced T memory cell response, particularly CD8^+^ T cell immunity, is also crucial for host protection. Our research evaluated the safety profile of *R. parkeri* 3A2 in both immunocompetent and immunocompromised mice. In C3H/HeN mice, potent efficacy was elicited by *R. parkeri* 3A2 without accumulating inflammatory cells, platelets or red blood cells, further highlighting that 3A2 is safe for immunocompetent individuals, at least by the stated vaccination usage. However, immunocompromised mice ultimately succumbed to both protective and challenge doses of *R. parkeri* 3A2, emphasizing that live-attenuated rickettsial vaccine may not be the option for individuals with severely impaired immunity.

In conclusion, we employed a live-attenuated candidate vaccine, which has been previously shown to confer complete protection against fatal rickettsioses, to demonstrate the immune correlation of serum antibodies with host protection. The neutralizing potency of vaccine-induced antibodies provided new and valuable insights into not only developing and evaluating the vaccine candidate but also generating novel therapeutic strategies for combating *R. parkeri* rickettsiosis.

## Methods

### Ethical Approval

Animal experiments were performed according to the NIH Guide for the Care and Use of Laboratory Animals (*National Research Council. 2011. Guide for the care and use of laboratory animals, 8th ed National Academies Press, Washington, DC.)* and approved by the University of Texas Medical Branch (UTMB) Institutional Animal Care and Use Committee. All the experiments described in this study were performed in a certified biosafety level 2 (BSL2) laboratory at UTMB. Tick saliva was prepared at the University of Southern Mississippi under the University of Southern Mississippi’s IACUC approved tick feeding on sheep under protocol # 15101501.6, with strict measures to minimize discomfort.

#### Rickettsiae

*Rickettsia parkeri* (Tate’s Hell strain) and *R. parkeri* mutant 3A2 were prepared as described previously^12^. In brief, WT *R. parkeri* was cultured and purified prior to electroporating in the presence of pLoxHimar plasmid DNA. pLoxHimar plasmid was constructed based on plasmid pCis Himar1 A7 by including mismatched lox5171 and lox2272 sites flanking the mCherry (red fluorescent marker) and *aadA* genes (encoding spectinomycin and streptomycin resistance). The insertion site of *R. parkeri* 3A2, RPATATE_0245::pLoxHimar, was determined by digestion with restriction enzymes followed by sequencing. *R. parkeri* 3A2 was cultured in the presence of 100 µg/ml of spectinomycin. Briefly, infected cells were placed on 32, 26, and 20% OptiPrep density gradient medium in a 6 X SPG buffer (218 mM sucrose, 3.76 mM KH_2_PO_4_, 7.1 mM K_2_HPO_4_, 4.9 mM potassium glutamate) bed (Sigma-Aldrich, St. Louis, MO) after sonication. As described previously^12,46^, *R. parkeri* were quantified by plaque assay or by real-time PCR with primers targeting rickettsial *gltA* gene and then stored at −80 °C until use.

### Ticks, tick saliva collection, and animals

Unfed adult Gulf Coast ticks *Amblyomma maculatum* were maintained at the University of Southern Mississippi in adherence to a standard protocol^47^. Before infesting ticks on a sheep, the adult ticks were kept at room temperature (RT) with approximately 90% humidity, following a photoperiod of 14 h of light and 10 h of darkness. Depending on the experimental design, adult ticks were fed on sheep for the required duration to facilitate subsequent saliva collection. Twenty partially engorged female ticks were removed from the sheep. These ticks were routinely checked for *R. parkeri* by PCR as described previously^48,49^. Tick saliva was obtained by stimulating partially blood-fed (5-7 days post-infestation) female *Amblyomma maculatum* to salivate into capillary tubes, employing the modified pilocarpine induction method outlined in a previous study^50^. The collected saliva was flash-frozen in liquid nitrogen and promptly stored at −80 °C for future use.

### Passive transfer of immune serum and rickettsial challenge

6 ~ 8-week-old female and male C3H/HeN mice and severely immunocompromised C3H-SCID mice were purchased from Jackson Laboratory (Bar Harbor, ME). Mice were maintained in the Institute’s Animal Facility under specific pathogen-free conditions in microisolator cages. C3H/HeN mice were i.d. immunized with a single dose of *R. parkeri* 3A2 at 1 × 10^3 copies of citrate synthase gene per mouse as described previously^12^. After 35 days of immunization, mouse sera were collected, and the titers of *R. parkeri*-specific IgG antibodies were determined by IFA using *R. parkeri* as antigen. Mice inoculated with PBS served as negative controls.

To determine whether immune sera conferred prophylactic protection against *R. parkeri* rickettsiosis, we passively transferred immune sera to B- and T-cell deficient C3H-SCID mice, followed by a lethal challenge. For preparation of the passive transfer, sera that had a *R. parkeri*-specific IgG antibody titer greater than 1:512 were pooled to normalize the dose that each C3H-SCID mouse would receive. Sera were diluted and filtered through a 0.22 µm pore size filter for sterilization. One day prior to and after the challenge with *R. parkeri*, each C3H-SCID mouse was i.p. injected with 100 µl *R. parkeri* 3A2- or PBS- immune sera diluted in PBS at 1:2 dilution. To determine whether immune sera confer protection against *R. parkeri* rickettsiosis in mice, C3H-SCID mice were challenged with *R. parkeri* via different inoculation routes after passive transfer of sera. A group of C3H-SCID mice were inoculated with *R. parkeri* i.v. through the tail vein at a lethal dose of 1 × 10^3 plaque forming units (PFU) per mouse. Another group of C3H-SCID mice were i.d. inoculated with *R. parkeri* at a lethal dose of 1 × 10^6 PFU, with or without 5 µl of tick saliva in a total inoculation volume of 50 µl. Mice inoculated with SPG served as controls. After the challenge, mice were monitored daily. Mouse body weight and illness score were recorded. In the present study, mice were anesthetized with an isoflurane variable-bypass vaporizer before intradermal inoculation with the indicated doses of rickettsiae. Animals that lost greater than 20% of their body weight were euthanized via CO₂ inhalation.

### Evaluation of *R. parkeri* 3A2-induced serum IgG antibodies against *R. parkeri* by IFA

The titers of IgG antibody specific against *R. parkeri* in mice immunized with a single dose of *R. parkeri* 3A2 were determined by IFA as described previously^12^. Briefly, *R. parkeri* were cultured in Vero cells and deposited on IFA slides. Sera of immunized and mock-immunized mice were prepared in 2-fold dilutions and overlaid on the slides. After incubation in a humidity chamber at 37 °C for 30 min, slides were washed three times and then dried. Bound antibody was detected with fluorescein-conjugated goat anti-mouse IgG (Vector Laboratories, Burlingame, CA, USA) diluted 1:500 in PBS containing 3% nonfat dry milk and 0.2% Evans blue (Millipore Sigma, St. Louis, MO, USA). The slides were washed and mounted with ProLong™ Gold Antifade Mountant with diamidino-2-phenylindole (DAPI, Invitrogen, Thermo Scientific, Waltham, MA, USA), and examined under an Echo Revolve Microscope equipped for epifluorescence. The results were judged positive if a specific green fluorescence was detected compared to the negative controls, and negative if no such fluorescence was found. According to the intensity of fluorescence, the results were classified as -, +, ++, +++, or ++++. The dilution of the sera, which showed the least fluorescence (example +) compared to the negative control, was considered the endpoint titer of IgG antibody specific to *R. parkeri*.

### Flow cytometric analysis of splenic plasma cells

Mouse spleen single-cell suspensions were prepared by mechanical dissociation and passage through a 70 μm nylon-strainer (352350, Corning). In spleen homogenates, red blood cells were removed using Red Blood Cell Lysis buffer (R7757, Sigma-Aldrich). Cell numbers were measured by hemocytometer, and live/dead staining was performed by using the Zombie UV™ Fixable Viability Kit as per manufacturer’s instructions (423107, BioLegend). After blocking the Fc-receptor with anti-mouse CD16/32 antibody (156604, BioLegend), cells were stained with fluorochrome-labeled antibodies as follows: CD19 with Brilliant Violet 711(115555, BioLegend), IgD with Brilliant Violet 510 (405723, BioLegend), CD45R/B220 with Alexa Fluor 700 (103232, BioLegend), and CD3 with Percp/cyanine 5.5 (100218, BioLegend). The staining was performed for 30 minutes in the dark at 4 °C. Cells were then washed with FACS buffer and were analyzed with a multiparametric flow cytometer FACS LSRFortessa (BD Biosciences). Data were analyzed using FlowJo software version 10.

### Quantification of *R. parkeri* by real-time PCR

Infected Vero cells or mouse tissues were collected in RNA*later* (Thermo Fisher Scientific, Waltham, MA), and the genomic DNA was extracted using a Qiagen DNA extraction kit (69506, Valencia, CA, USA) as per the manufacturer’s instructions. Quantitative real-time PCR was performed using an iCycler (Bio-Rad, Hercules, CA, USA) to determine the concentrations of rickettsiae as described previously^12,46^. In brief, extracted genomic DNA was amplified by primers and TaqMan probes for *Rickettsia*-specific citrate synthase (CS)-encoding gene (*gltA*), as described in our previous studies: *gltA* forward, GAGAGAAAATTATATCCAAATGTTGAT; *gltA* reverse, AGGGTCTTCGTGCATTTCTT; *gltA* probe, CATTGTGCCATCCAGCCTACGGT. The *gltA* probe was labeled with 6-carboxyfluorescein (FAM). Two-step cycle parameters (95 °C and 60 °C) were used. The results were normalized to the amount (in micrograms) of genomic DNA in the same sample and expressed as CS gene copy number per microgram of genomic DNA.

For the molecular quantification of viable rickettsiae in a stock, a 1:100 dilution of rickettsial stock vial was added to each well of a 6-well plate containing a confluent monolayer of Vero cells. The plate was centrifuged at 800× g for 5 min and then incubated at RT for one hour. Next, cells were washed three times with PBS and then incubated at 37 °C in Dulbecco’s Modified Eagle Medium (DMEM) containing 10% fetal bovine serum (FBS) for another hour. After washing again with PBS, cells were collected and processed for real-time PCR to quantify viable rickettsiae that had entered Vero cells as described above. The copies of the CS gene in rickettsial stock were used to calculate the multiplicity of infection (MOI) in in vitro infection of mammalian host cells.

### Hematological analysis

Whole blood samples were collected from mice on day 15 post immunization in potassium salt of ethylenediaminetetraacetic acid (K_2_EDTA) tubes (365974, BD Biosciences) and used for the analysis of lymphocytes, neutrophils, white blood cells, monocytes, hematocrit, and platelets. The analyses were performed on a VetScan HM5 hematology system (Abaxis).

### Histopathology

Liver tissues were harvested from uninfected and infected mice and immediately stored in 10% buffered formalin. Specimens were embedded in paraffin, sectioned at 5 μm thickness, and stained with hematoxylin-eosin (H&E). Sections were imaged at ×10 and ×40 under bright field using the Keyence all-in-one fluorescent microscope (Keyence, BZ-X810), and at least ten random fields were captured for each section. The images were captured, examined, and analyzed for inflammatory foci in a quantitative manner.

### Measurements of serum IFN-γ levels

The amount of IFN-γ in mouse sera was measured using a mouse IFN-γ enzyme-linked immunosorbent assay (ELISA) kit (88-7314-88, Thermo Fisher Scientific) following the instructions provided by the manufacturer. Briefly, each well in a 96-well micro-plate was coated with anti-mouse IFN-γ capture antibody overnight at 4 °C. After three washes with wash buffer (PBS containing 0.05% Tween-20), each well was blocked for 1 hour at RT with ELISA/ELISPOT diluent (1X). After three washes, 100 µl of diluted serum samples were added. The standard curve was created using various concentrations of recombinant IFN-γ, diluted in ELISA/ELISPOT diluent, with final concentrations ranging from 15.62 to 2000 pg/ml. Following an overnight incubation at 4 °C, the plate was washed three times with wash buffer, and 100 µl of anti-mouse IFN-γ detection antibody was added in each well. The plate was then incubated at RT for an hour. After washes, each well was incubated with 100 µl of streptavidin-horseradish peroxidase (HRP) for 30 minutes. After incubation, the plate was washed five times, and 100 µl of 3,3’,5,5’-Tetramethylbenzidine (TMB) solution was added to each well. After incubating the plate at RT for 15 minutes, 100 µl of stop solution (N600, Thermo Fisher Scientific) was added to each well, and the absorbance at 450 nm was measured using the SpectraMax iD5 microplate reader (Molecular Devices, Sunnyvale, CA). The concentration of IFN-γ (pg/ml) in serum samples was analyzed, and the sensitivity of this assay was 16 pg/ml.

### Plaque assay

Vero cells were seeded on a 24-well plate. A series of 10-fold dilutions of rickettsiae, in 200 μL of DMEM containing 10% FBS, were added to each well containing a confluent monolayer of Vero cells. After cells were incubated with rickettsiae at RT for 6 h, they were placed at 37 °C with 5% CO_2_ overnight before a semi-solid agarose gel overlay was applied on top of the infected Vero cells. After the gel was solidified at RT, the plate was incubated at 37 °C with 5% CO_2_. After 3–5 days, the plaques were quantified.

### Plaque reduction neutralization assay

To investigate whether IgG antibodies of live-attenuated *R. parkeri* 3A2-induced mouse sera inhibit the infectivity of rickettsiae by preventing their binding to mammalian host cells, we determined the neutralizing activity specific against WT *R. parkeri* by plaque reduction assay using Vero cells. Sera were collected from *R. parkeri* 3A2 or mock-immunized mice 8 weeks after immunization. Pooled mouse sera were heated at 56 °C for 1 h to inactivate the complement, followed by dilution in a 2-fold series from 1:100 to 1:3200. Diluted sera were incubated with an equal volume of *R. parkeri* WT at 37 °C for 2 h and subsequently added to the confluent monolayer of Vero cells in a 24-well cell culture plate at a volume of 200 µL per well. The plates were first incubated at RT for 6 h and then placed at 37 °C with 5% CO_2_ overnight. On the next day, cells in each well were overlaid with 1 mL of modified Eagle’s medium (MEM; Gibco) containing 0.75% low melting agarose (50100, Lonza) and 2% fetal bovine serum (Gibco). After the gel was solidified, plates were incubated at 37 °C with 5% CO_2_. Cytopathic effect was monitored daily by the Revolve microscope (Echo Inc., San Diego, CA, USA). After 4 days, the cells were fixed in 4% (v/v) paraformaldehyde (1 ml per well) at RT for 30 min. Then, the cells were washed with PBS and stained in 1% crystal violet solution. After three washes with water, plaques were observed and counted. In parallel, Vero cells incubated with the same volume of a mixture containing 100 µL of rickettsiae and 100 µL of medium or only medium served as positive and negative controls.

The average number of plagues generated by mock-immune sera for each dilution was calculated. The percentage of reduced plaques neutralized by immune sera was calculated as: (1 – number of plaques in immune sera/average number of plaques in mock immune sera) × 100. The neutralizing titer was defined as the serum dilution that reduced plaques by at least 50% compared with those observed with sera from mock-immunized controls.

Additionally, to determine the exact antibody titer achieving 50% neutralization based on the neutralization curve, sets of 3-4 technical replicates were run for each of three independent experiments. Percent reduction in *R. parkeri* plaques was calculated for each technical replicate of each independent experiment as compared to the mean plaque counts from four no-serum control wells. Serum dilution factors were log_10_ transformed prior to analysis. The mean percent reduction at a given serum dilution for each of the three independent experiments was analyzed by the variable slope log (agonist) vs. response function, with the upper and lower bounds constrained to 100 and 0, respectively. The resulting EC50 values and overall curves were compared by the extra sum-of-squares F test. All analysis was done in Prism v10.4.1 (GraphPad, La Jolla, CA).

### Mouse serum antibody isotype profile

For isotype ELISA, the 96-Well micro plates (161093, Thermo Fisher Scientific) were coated overnight at 4 °C with 3 µg of WT *R. parkeri* antigen per well in 100 µL PBS (pH 7). Plates were washed three times with PBS containing 0.05% Tween-20 (PBSTW). Plates were then blocked with 100 µL of blocking buffer (0.1% Tween-20, 2% BSA, 1x PBS) for 1 h at RT and then washed as described above. Serum samples were diluted at two fold dilutions from 1:100 in PBS, and log2 serial dilutions were added to the plates, followed by overnight incubation at 4 °C. Isotype-specific detection was performed by 1 h of incubation with horseradish peroxidase (HRP)-conjugated monoclonal antibodies to mouse IgG (1030-05, SouthernBiotech), IgG1 (1071-05, SouthernBiotech), IgG2a (1080-05, SouthernBiotech), IgG2b (1091-05, SouthernBiotech), IgG3 (1101-05, SouthernBiotech), or IgA (1040-05, SouthernBiotech) diluted 1:5000 in 3% BSA. After incubation, plates were washed five times with wash buffer and incubated with 100 µL of TMB substrate solution (34028, Thermo Fisher Scientific). The plates were incubated at RT for 20 min. Absorbance was read at 650 nm using the SpectraMax iD5 microplate reader (Molecular Devices, Sunnyvale, CA) after the addition of 100 µl of stop solution (5150-0023, SeraCare). The results were reported as the reciprocal of the highest titer giving an optical density (O.D.) reading of at least mean +2 SD greater than the sera from mice inoculated with PBS. All assays were performed in triplicate, and results were shown as the mean reciprocal endpoint titer.

### Statistical analysis

One-way analysis of variance (ANOVA) with Bonferroni’s procedure was used for the comparison of multiple experimental groups and analyzed statistically with GraphPad Prism software version 9.1.1 (GraphPad Software, San Diego, CA, USA). A t-test was used to compare the outcomes between two groups at a specific time point(s). *p* values of 0.05 or less were considered significant. Survival differences were compared using Kaplan-Meier survival curves, followed by a log rank test.

Two-way ANOVA and the Friedman test were used to evaluate differences in outcome growth between the two experimental groups, considering the main effects of group and day, as well as their interaction.

## Supplementary information

Supplementary information

## Data Availability

All datasets generated and analyzed during this study are included in the manuscript and supplementary files. The original raw data can also be acquired from the corresponding author upon request.

## References

[1] Hechemy, K. E. et al. Discrepancies in Weil-Felix and microimmunofluorescence test results for Rocky Mountain spotted fever. *J. Clin. Microbiol.* **9**, 292–293 (1979). 107194 10.1128/jcm.9.2.292-293.1979 PMC273012

[2] Paddock, C. D., Lane, R. S., Staples, J. E. & Labruna, M. B. *Changing paradigms for tick-borne diseases in the Americas*. National Academies Press, 2016.

[3] Biggs, H. M. et al. Diagnosis and management of tickborne rickettsial diseases: rocky mountain spotted fever and other spotted fever group rickettsioses, ehrlichioses, and anaplasmosis - United States. *MMWR Recomm. Rep.* **65**, 1–44 (2016). 10.15585/mmwr.rr6502a1 27172113

[4] Paddock, C. D. et al. *Rickettsia parkeri*: a newly recognized cause of spotted fever rickettsiosis in the United States. *Clin. Infect. Dis.* **38**, 805–811 (2004). 10.1086/381894 14999622

[5] Blanton, L. S. The Rickettsioses: a practical update. *Infect. Dis. Clin. North Am.* **33**, 213–229 (2019). 30712763 10.1016/j.idc.2018.10.010 PMC6364315

[6] Demma, L. J. et al. Rocky Mountain spotted fever from an unexpected tick vector in Arizona. *N. Engl. J. Med.* **353**, 587–594 (2005). 16093467 10.1056/NEJMoa050043

[7] de Oliveira, S. V. et al. An update on the epidemiological situation of spotted fever in Brazil. *J. Venom. Anim. Toxins Incl. Trop. Dis.* **22**, 22 (2016). 27555867 10.1186/s40409-016-0077-4 PMC4994305

[8] Alvarez-Hernandez, G. et al. Rocky Mountain spotted fever in Mexico: past, present, and future. *Lancet Infect. Dis.* **17**, e189–e196 (2017). 28365226 10.1016/S1473-3099(17)30173-1

[9] Azad, A. F. Pathogenic rickettsiae as bioterrorism agents. *Clin. Infect. Dis.* **45**, S52–S55 (2007). 17582570 10.1086/518147

[10] Walker, D. H. The realities of biodefense vaccines against *Rickettsia*. *Vaccine* **27**, D52–D55 (2009). 10.1016/j.vaccine.2009.07.045 PMC2909128 19837287

[11] Richards, A. L. Rickettsial vaccines: the old and the new. *Expert Rev. Vaccines* **3**, 541–555 (2004). 15485334 10.1586/14760584.3.5.541

[12] Arroyave, E. et al. *Rickettsia parkeri* with a genetically disrupted phage integrase gene exhibits attenuated virulence and induces protective immunity against fatal rickettsioses in mice. *Pathogens* **10**, 819 (2021). 10.3390/pathogens10070819 PMC8308654 34208806

[13] Zhang, J. Z., Hao, J. F., Walker, D. H. & Yu, X. J. A mutation inactivating the methyltransferase gene in avirulent Madrid E strain of *Rickettsia prowazekii* reverted to wild type in the virulent revertant strain Evir. *Vaccine* **24**, 2317–2323 (2006). 10.1016/j.vaccine.2005.11.044 16364512

[14] Kenyon, R. H., Sammons, L. S. & Pedersen, C. E. Jr Comparison of three rocky mountain spotted fever vaccines. *J. Clin. Microbiol.* **2**, 300–304 (1975). 810494 10.1128/jcm.2.4.300-304.1975 PMC362799

[15] Clements, M. L. et al. Reactogenicity, immunogenicity, and efficacy of a chick embryo cell-derived vaccine for Rocky Mountain spotted fever. *J. Infect. Dis.* **148**, 922–930 (1983). 6415182 10.1093/infdis/148.5.922

[16] Riley, S. P., Patterson, J. L. & Martinez, J. J. The rickettsial OmpB beta-peptide of Rickettsia conorii is sufficient to facilitate factor H-mediated serum resistance. *Infect. Immun.* **80**, 2735–2743 (2012). 22615250 10.1128/IAI.00349-12 PMC3434587

[17] Crocquet-Valdes, P. A. et al. Immunization with a portion of rickettsial outer membrane protein A stimulates protective immunity against spotted fever rickettsiosis. *Vaccine* **20**, 979–988 (2001). 11738766 10.1016/s0264-410x(01)00377-2

[18] Diaz-Montero, C. M., Feng, H. M., Crocquet-Valdes, P. A. & Walker, D. H. Identification of protective components of two major outer membrane proteins of spotted fever group Rickettsiae. *Am. J. Trop. Med. Hyg.* **65**, 371–378 (2001). 11693887 10.4269/ajtmh.2001.65.371

[19] Riley, S. P. et al. Failure of a heterologous recombinant Sca5/OmpB protein-based vaccine to elicit effective protective immunity against *Rickettsia rickettsii* infections in C3H/HeN mice. *Pathog. Dis.* **73**, ftv101 (2015). 10.1093/femspd/ftv101 PMC4732028 26519448

[20] Chan, Y. G., Riley, S. P., Chen, E. & Martinez, J. J. Molecular basis of immunity to rickettsial infection conferred through outer membrane protein B. *Infect. Immun.* **79**, 2303–2313 (2011). 21444665 10.1128/IAI.01324-10 PMC3125829

[21] Burke, T. P. et al. Interferon receptor-deficient mice are susceptible to eschar-associated rickettsiosis. *Elife* **10**, e67029 (2021). 10.7554/eLife.67029 PMC8428839 34423779

[22] Walker, D. H. & Dumler, J. S. The role of CD8 T lymphocytes in rickettsial infections. *Semin Immunopathol.* **37**, 289–299 (2015). 25823954 10.1007/s00281-015-0480-x PMC4458380

[23] Feng, H. M., Whitworth, T., Popov, V. & Walker, D. H. Effect of antibody on the rickettsia-host cell interaction. *Infect. Immun.* **72**, 3524–3530 (2004). 15155660 10.1128/IAI.72.6.3524-3530.2004 PMC415703

[24] Feng, H. M., Walker, D. H. & Wang, J. G. Analysis of T-cell-dependent and -independent antigens of *Rickettsia conorii* with monoclonal antibodies. *Infect. Immun.* **55**, 7–15 (1987). 10.1128/iai.55.1.7-15.1987 PMC260273 3793235

[25] Li, H., Lenz, B. & Walker, D. H. Protective monoclonal antibodies recognize heat-labile epitopes on surface proteins of spotted fever group rickettsiae. *Infect. Immun.* **56**, 2587–2593 (1988). 2458318 10.1128/iai.56.10.2587-2593.1988 PMC259616

[26] Wang, P. et al. Th1 epitope peptides induce protective immunity against *Rickettsia rickettsii* infection in C3H/HeN mice. *Vaccine* **35**, 7204–7212 (2017). 10.1016/j.vaccine.2017.09.068 29032899

[27] Walker, D. H., Popov, V. L., Wen, J. & Feng, H. M. *Rickettsia conorii* infection of C3H/HeN mice. A model of endothelial-target rickettsiosis. *Lab Invest* **70**, 358–368 (1994). 7511715

[28] Iversen, J. O., Spalatin, J., Fraser, C. E. & Hanson, R. P. Ocular involvement with *Chlamydia psittaci* (strain M56) in rabbits inoculated intravenously. *Can. J. Comp. Med.* **38**, 298–302 (1974). PMC1319872 4277591

[29] Levy, N. J. & Moulder, J. W. Attachment of cell walls of *Chlamydia psittaci* to mouse fibroblasts (L cells). *Infect. Immun.* **37**, 1059–1065 (1982). 10.1128/iai.37.3.1059-1065.1982 PMC347648 7129628

[30] Britto, C. & Alter, G. The next frontier in vaccine design: blending immune correlates of protection into rational vaccine design. *Curr. Opin. Immunol.* **78**, 102234 (2022). 35973352 10.1016/j.coi.2022.102234 PMC9612370

[31] Casadevall, A. Antibody-based vaccine strategies against intracellular pathogens. *Curr. Opin. Immunol.* **53**, 74–80 (2018). 29704764 10.1016/j.coi.2018.04.011 PMC6245945

[32] Mackin, S. R., Sariol, A. & Diamond, M. S. Antibody-mediated control mechanisms of viral infections. *Immunol. Rev.* **328**, 205–220 (2024). 39162394 10.1111/imr.13383 PMC11661935

[33] Burton, D. R. Antiviral neutralizing antibodies: from in vitro to *in vivo* activity. *Nat. Rev. Immunol.* **23**, 720–734 (2023). 10.1038/s41577-023-00858-w PMC10108814 37069260

[34] Osterloh, A. Vaccination against bacterial infections: challenges, progress, and new approaches with a focus on intracellular bacteria. *Vaccines* **10**, 751 (2022). 10.3390/vaccines10050751 PMC9144739 35632507

[35] He, R. T. et al. Antibodies that block virus attachment to Vero cells are a major component of the human neutralizing antibody response against dengue virus type 2. *J. Med. Virol.* **45**, 451–461 (1995). 7666046 10.1002/jmv.1890450417

[36] Sanapala, S., Rahav, H., Patel, H., Sun, W. & Curtiss, R. Multiple antigens of *Yersinia pestis* delivered by live recombinant attenuated Salmonella vaccine strains elicit protective immunity against plague. *Vaccine* **34**, 2410–2416 (2016). 10.1016/j.vaccine.2016.03.094 PMC5484397 27060051

[37] Hessel, A. et al. A pandemic influenza H1N1 live vaccine based on modified vaccinia Ankara is highly immunogenic and protects mice in active and passive immunizations. *PLoS One* **5**, e12217 (2010). 20808939 10.1371/journal.pone.0012217 PMC2922371

[38] Feunou, P. F., Bertout, J. & Locht, C. T- and B-cell-mediated protection induced by novel, live attenuated pertussis vaccine in mice. Cross protection against parapertussis. *PLoS One* **5**, e10178 (2010). 20419113 10.1371/journal.pone.0010178 PMC2855369

[39] Asano, K. et al. Passive immunization with anti-ActA and anti-listeriolysin O antibodies protects against Listeria monocytogenes infection in mice. *Sci. Rep.* **6**, 39628 (2016). 28004800 10.1038/srep39628 PMC5177876

[40] Lu, L. L., Suscovich, T. J., Fortune, S. M. & Alter, G. Beyond binding: antibody effector functions in infectious diseases. *Nat. Rev. Immunol.* **18**, 46–61 (2018). 29063907 10.1038/nri.2017.106 PMC6369690

[41] Giuntini, S. et al. Human IgG1, IgG3, and IgG3 hinge-truncated mutants show different protection capabilities against meningococci depending on the target antigen and epitope specificity. *Clin. Vaccin. Immunol.* **23**, 698–706 (2016). 10.1128/CVI.00193-16 PMC4979173 27307451

[42] Nimmerjahn, F., Vidarsson, G. & Cragg, M. S. Effect of posttranslational modifications and subclass on IgG activity: from immunity to immunotherapy. *Nat. Immunol.* **24**, 1244–1255 (2023). 37414906 10.1038/s41590-023-01544-8

[43] Dahmani, M., Cook, J. H., Zhu, J. C. & Riley, S. P. Contribution of classical complement activation and IgM to the control of Rickettsia infection. *Mol. Microbiol* **116**, 1476–1488 (2021). 34725868 10.1111/mmi.14839 PMC8955150

[44] Raoult, D. & Dasch, G. A. Immunoblot cross-reactions among Rickettsia, Proteus spp. and Legionella spp. in patients with Mediterranean spotted fever. *FEMS Immunol. Med. Microbiol.* **11**, 13–18 (1995). 7541270 10.1111/j.1574-695X.1995.tb00073.x

[45] Damelang, T. et al. Impact of structural modifications of IgG antibodies on effector functions. *Front. Immunol.* **14**, 1304365 (2023). 38259472 10.3389/fimmu.2023.1304365 PMC10800522

[46] Bechelli, J. et al. MyD88 mediates instructive signaling in dendritic cells and protective inflammatory response during rickettsial infection. *Infect. Immun.* **84**, 883–893 (2016). 26755162 10.1128/IAI.01361-15 PMC4807495

[47] Patrick, C. D. & Hair, J. A. Laboratory rearing procedures and equipment for multi-host ticks (Acarina: Ixodidae). *J. Med. Entomol.* **12**, 389–390 (1975). 1181449 10.1093/jmedent/12.3.389

[48] Budachetri, K. et al. The tick endosymbiont Candidatus Midichloria mitochondrii and selenoproteins are essential for the growth of Rickettsia parkeri in the Gulf Coast tick vector. *Microbiome* **6**, 141 (2018). 30103809 10.1186/s40168-018-0524-2 PMC6090677

[49] Budachetri, K. et al. An insight into the microbiome of the Amblyomma maculatum (Acari: Ixodidae). *J. Med. Entomol.* **51**, 119–129 (2014). 24605461 10.1603/me12223 PMC3956751

[50] Crispell, G. et al. Discovery of alpha-gal-containing antigens in North American tick species believed to induce red meat allergy. *Front. Immunol.* **10**, 1056 (2019). 31156631 10.3389/fimmu.2019.01056 PMC6533943

