# Supplementary material for: Vaccination-induced neutralizing antibodies in immunocompetent hosts correlate with protection against rickettsiae
Source: NPJ Vaccines. 2025 Aug 1;10:180. doi: 10.1038/s41541-025-01228-4 (PMC12316887; doi:10.1038/s41541-025-01228-4)
Supplement: Supplementary file 1 — Supplementary information [file 41541_2025_1228_MOESM1_ESM.pdf]

Supplementary Fig 1

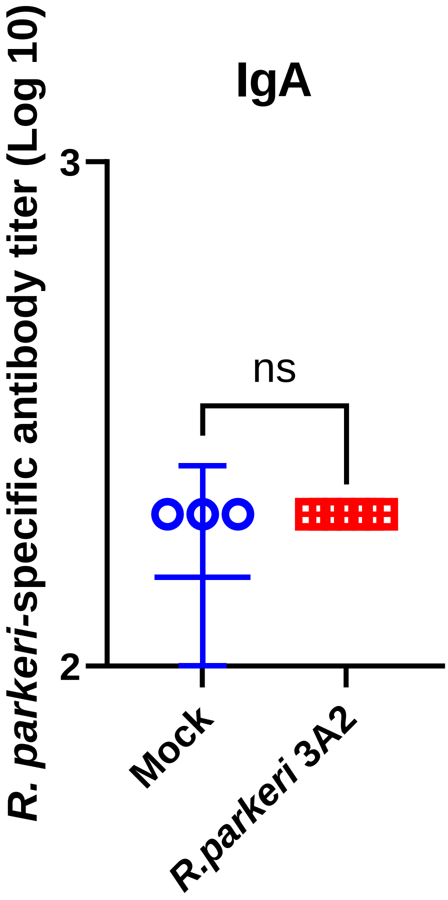

S1. Non-detectable levels of IgA antibodies specific against WT *R. parkeri* in sera of *R. parkeri* 3A2-immunized mice. C3H/HeN mice were immunized with a single dose of *R. parkeri* 3A2 i.d.. Mice immunized the PBS served as mock controls. Sera were collected after 56 days of immunization. The titers of WT *R. parkeri*-specific IgA antibodies in immune sera were determined by ELISA as described in the corresponding section of Materials and Methods. Each group include 4-6 mice. ns, not statistically significant.
